# Supplementary material for: Plant Growth-Promoting Bacteria from Tropical Soils: In Vitro Assessment of Functional Traits
Source: Microorganisms. 2025 Oct 7;13(10):2321. doi: 10.3390/microorganisms13102321 (PMC12566490; doi:10.3390/microorganisms13102321)
Supplement: Supplementary file 1 [file microorganisms-13-02321-s001.zip › microorganisms-3877458-supplementary.pdf]

Supplementary Materials

Table S1. Code, taxonomic identification (by MALDI-TOF MS), score values, and functional characterization of bacterial isolates. Traits include solubilization of thermophosphate, aluminum phosphate, and phonolite (expressed as released P or K in mg·L⁻¹, with corresponding pH); auxin production (µg·mL⁻¹); ACC deaminase activity (presence indicated by +); siderophore production and type (e.g., carboxylate); and antagonistic activity against phytopathogens (percentage of inhibition).

Code	Identification	Score	Termophosphate		Aluminum phosphate		Phonolite		Auxin	ACC	Siderophore	Antagon
			**P (mg.L^{-1})**	**pH**	**P (mg.L^{-1})**	**pH**	**K (mg.L^{-1})**	**pH**	**µg.mL^{-1}**			**%In**
SS11	*Enterobacter* sp.	1.66	26.9	8.6	10.5	3.5	23.5	6.0	1.34	-	-	0
SS15	*Enterobacter hormaechei*	2.20	25.3	7.0	9.6	3.6	22.4	4.6	6.18	-	Carboxylate	15
SS17	*Bacillus cereus*	2.15	42.5	5.3	5.9	3.8	14.2	4.3	6.76	-	Carboxylate	5
SS18	*Bacillus cereus*	2.19	45.2	5.3	5.2	4.8	12.0	4.6	5.39	-	Carboxylate	0
SS26	*Bacillus cereus*	2.27	24.5	6.9	5.3	4.6	14.1	4.8	1.53	-	-	15
SS28	*Bacillus cereus*	2.41	29	5.3	1.2	4.5	12.6	4.6	1.92	-	Carboxylate	16
SS29	*Bacillus cereus*	2.13	31.3	5.3	5.2	4.8	12.8	4.4	3.3	-	Carboxylate	4
SS31	*Bacillus cereus*	2.31	24.2	6.0	3.5	4.7	14.8	4.6	1.43	-	-	15
SS33	*Bacillus cereus*	2.29	26	5.3	3.6	5.2	14.9	5.2	5.95	-	-	0
SS35	*Bacillus cereus*	2.31	20.1	7.1	4.7	4.8	3.9	5.1	6.85	-	-	16
SS36	*Bacillus cereus*	2.49	34.4	5.8	8.0	4.7	7.3	5.2	2.01	-	Carboxylate	18
SS68	*Bacillus cereus*	2.13	36.9	5.4	1.6	4.7	2.8	5.1	5.58	-	-	20
SS80	*Bacillus cereus*	2.44	39.9	5.8	4.0	4.2	2.9	5.2	2.54	-	-	0
SS88	*Bacillus cereus*	2.30	19.8	5.5	2.7	4.8	16.4	5.2	1.56	-	-	0
SS89	*Bacillus cereus*	2.09	24.0	8.3	3.4	3.5	15.5	5.2	5.86	-	-	28
SS101	*Bacillus cereus*	2.27	34.2	5.5	7.6	4.7	9.2	6.4	6.48	-	-	44
SS107	*Bacillus cereus*	2.05	27.1	6.5	7.4	4.5	5.3	5.9	5.07	-	Carboxylate	0
SS120	*Staphylococcus sciuri*	1.99	26.8	6.4	3.7	3.8	21.7	5.5	9.65	-	-	15
SS137	*Bacillus cereus*	2.32	22.6	6.1	5.8	4.4	8.1	5.4	3.39	-	Carboxylate	52
SS138	*Bacillus cereus*	2.37	22.1	6.5	3.4	5.0	9.5	5.2	3.23	-	Carboxylate	11
SS145	*Enterobacter*	2.44	18.8	6.4	3.2	3.8	20.1	6.3	4.41	-	Carboxylate	30

	hormaechei											
SS150	*Pantoea* sp.	2.41	16.3	6.1	0.8	3.8	22.4	5.7	7.68	-	-	8
SS183	*Pseudomonas aeruginosa*	2.45	18.9	7.1	4.5	4.8	22.8	5.3	5.98	+	Carboxylate	91
SS186	*Enterobacter hormaechei*	2.38	14.4	6.0	2.8	3.8	23.5	5.4	8.02	-	Carboxylate	18
SS246	*Bacillus cereus*	2.14	25.6	6.4	9.3	4.5	7.5	5.4	3.97	-	-	27
SS249	*Bacillus cereus*	2.36	24.1	6.0	2.3	4.7	3.6	5.3	2.92	-	Carboxylate	9
CCT 3116	*Bacillus altitudinis*	x	30.9	5.7	1.4	4.3	13.3	5.0	4.2	-	Carboxylate	4
Bp2	*Bacillus pumillus*	1.83	10.4	6.2	3.7	4.5	10.6	5.7	6.02	-	Carboxylate	0
Bti	*Bacillus thunrigiensis*	2.00	27.3	5.6	5.1	3.9	10.4	5.5	6.53	-	Carboxylate	0
K22	*Pseudomonas azotoformans*	1.78	36.5	5.0	16.6	4.0	16.7	4.2	8.94	-	Carboxylate	0
BR 11281	*Gluconacetobacter diazotrophicus*	x	19.9	5.7	50.6	2.9	x	x	x	x	x	x
BR 11001	*Azospirillum brasilense*	x	x	x	x	x	x	x	29.4	x	x	x
BR 11790	*Herbaspirillum seropedicae*	x	x	x	x	x	x	x	x	+	x	x

ACC: ACC-deaminase; Antagon: antagonistic activity; %In: percentage inhibition; BR 11281 was used as the positive control for phosphorus solubilization; BR 11001 for auxin production; and BR 11790 for ACC deaminase production. The symbols denote: (+) present, (-) absent, (x) not tested.
